# Interaction of genetic markers associated with serum alkaline phosphatase levels in the Japanese population

**DOI:** 10.1038/hgv.2015.19

**Published:** 2015-07-02

**Authors:** Masatoshi Masuda, Kayo Okuda, Daisuke D Ikeda, Haretsugu Hishigaki, Tsutomu Fujiwara

**Affiliations:** 1 Department of Clinical Research and Development, Otsuka Pharmaceutical, Osaka, Japan; 2 Institute of Biomedical Innovation, Otsuka Pharmaceutical, Tokushima, Japan

## Abstract

In the present genome-wide association study of 2,994 Japanese subjects, rs2071699 (35C>T) in the fucosyltransferase 1 (*FUT1*) gene was identified as a marker associated with serum alkaline phosphatase (ALP) levels. This gene encodes α(1,2)-fucosyltransferase, which is responsible for the synthesis of H antigens. In a linear regression model incorporating genetic markers, rs550057 (C>T), which is located within an intron of the ABO blood group (*ABO*) locus, rs2071699 in *FUT1* and a gene–gene interaction between these loci accounted for 12.4, 0.9 and 0.3% of the total variability in the serum ALP level, respectively. Further association analysis using imputed genotypes detected rs1047781 in *FUT2*. rs1047781 is well known in this association with serum ALP levels and showed a moderate linkage with rs2071699 in *FUT1*. An interaction analysis using rs1047781 in *FUT2* also suggested that the interaction with rs550057 in *ABO* is significant and contributes to the interindividual variance of serum ALP levels as well as rs2071699 in the *FUT1* gene. Thus, there is evidence of interactions between *ABO* and *FUT1*/*FUT2* on serum ALP levels, regardless of the possibility that rs2071699 in *FUT1* is a proxy of rs1047781 in *FUT2* in the Japanese population.

## Introduction

Alkaline phosphatases (ALPs) catalyze the hydrolysis of organic phosphate esters in an alkaline environment and are highly expressed in liver, bone, intestine and placenta.^1^ In healthy subjects, serum ALP is mainly derived from the liver, bone, kidney and partially from the intestine. Age, sex, smoking, diet, body mass index (BMI) and physical activity can affect serum ALP levels.^2^ Intestinal ALP in the serum is associated with the ABO blood serotype and secretor phenotype.^3,4^ As opposed to non-secretors, secretors express the ABO antigen in their saliva, and intestinal ALP levels measured in the serum are higher in secretors than in non-secretors. In secretors, differences in serum ALP levels are present among the ABO blood serotypes; for example, the serum ALP level is higher in individuals with the ABO blood serotype B or O than in those with serotype A or AB. This difference occurs because blood type A erythrocytes bind almost all intestinal ALP in the serum, whereas blood type B or O erythrocytes bind a lower proportion of serum ALP.^5,6^ The ABO blood serotype and secretor phenotype are determined by variants in the ABO blood (*ABO*) gene and the fucosyltransferase-2 (*FUT2*) gene, respectively.^7,8^

Several genome-wide association studies (GWASs) have shown strong associations of single-nucleotide polymorphisms (SNPs) in the *ABO* gene with ALP in populations of Chinese, Japanese and European ancestry.^9–11^ Many other loci, including *FUT2,* have also been identified in GWASs.^9–11^ The measurement of serum ALP involves one liver function test that is frequently performed as a part of routine health checks. In addition, the serum ALP level is associated with biliary diseases and tumors of the hepatobiliary system.^12^ Thus, it is important to clarify the genetic factors that influence variations in serum ALP levels in healthy subjects.

In this study, we explored loci associated with serum ALP levels in a healthy Japanese population and identified an SNP in the *FUT1* gene as a new marker associated with ALP levels. We then evaluated the contribution of this SNP to interindividual variations in serum ALP levels.

## Materials and methods

### Genotype data and quality control

Genome-wide genotype data from 2,994 healthy Japanese subjects were obtained from the Japan PGx Data Science Consortium (http://www.jpdsc.org/english/) database. The Japanese subjects recruited in this study all had parents and grandparents who were also Japanese. The inclusion criteria for the subjects were as follows: (1) over 20 years of age at the time of informed consent; (2) healthy Japanese adult residing in Japan; and (3) received adequate explanations of the purpose and contents of the study, volunteered of their own will and gave a written consent to participate in the study. The exclusion criteria were as follows: (1) persons with cardiovascular disease, kidney disease, liver disease or some other condition considered to render them ineligible for the study; (2) those with a blood relative within the third degree of kinship who had already participated in the study; (3) past participants in the study; and (4) persons whom the principal investigators judged to be ineligible to participate. Informed consent was obtained from all subjects for the use of their DNA for association studies, and the ethical committee of Japan PGx Data Science Consortium approved the DNA sampling study. The characteristics of the study population are shown in Supplementary Table 1.

The genotype data included 2,379, 855 SNPs analyzed using an Illumina HumanOmni 2.5–8 platform (San Diego, CA, USA). For quality control, SNPs with call rates of <99%, minor allele frequencies (MAFs) of <0.01, and Hardy–Weinberg equilibrium values of <5.0×10^−8^ (Fisher’s exact test) were excluded from the GWAS.

### Serum ALP

The serum ALP levels in subjects who fasted overnight were measured using standard laboratory procedures. Log-transformed values of these levels were used for association analysis. The ALP data from ten subjects were excluded from the GWAS because their log-transformed ALP values were not within the mean log-transformed ALP±4 s.d. or because their body weight and height were not obtained. Finally, the serum ALP values and genotype data from 2,984 subjects were used for the GWAS.

### Genome-wide association study for ALP

Genome-wide association with serum ALP levels was assessed via linear regression analysis, assuming an additive genetic model with the following five covariates: age; sex; BMI; and the top two eigenvectors obtained by principal component analysis. Principal component analysis was performed for tag-SNPs excluding the human leukocyte antigen region on chromosome 6p21.1-p21.3, because a significant level of the Japanese population structure was previously determined from the human leukocyte antigen genotype data.^13^ The genome-wide association statistical level was set to 5×10^−8^.

### Multiple SNP association analysis

To evaluate the contribution of genetic factors to the interindividual variability in serum ALP levels, linear regression analysis was performed with stepwise model selection using Akaike’s information criterion.^14^ Two significant SNPs, rs550057 in the *ABO* gene and rs2071699 in the *FUT1* gene, were incorporated as genetic markers into a linear regression model with the covariates of age, sex and BMI. We examined the additive effect and the deviation from additivity (dominance deviation) at each SNP according to the method described by Saito *et al*.^15^ The additive effect was coded as 0, 1 or 2 for three genotypes according to the number of T allele for each SNP, and the dominance deviation was coded as 0 and 1 for homozygotes and heterozygotes, respectively.

### Gene–gene interaction analysis

We evaluated the effects of the interaction between rs2071699 in the *FUT1* gene and rs550057 in the *ABO* gene on the serum ALP level. In the multiple SNP association analysis described above, the mode of inheritance was estimated to be T-allele dominant for rs550057 and additive for rs2071699. Then, in a gene–gene interaction analysis between the two SNPs, genotypes for rs550057 were coded for 0, 1 and 1 for CC, CT and TT, respectively. The genotypes for rs2071699 were coded for 0, 1 and 2 according to the number of T-alleles. The interaction term between rs2071699 and rs550057 was added as a variable with the covariates of age, sex and BMI.

For the gene–gene interaction, we also evaluated the effect of the rs2071699 genotype on serum ALP levels for each stratum of the rs550057 genotypes (CC, CT/TT) using a linear regression model for log-transformed ALP. The significance level was set to 0.025 (0.05/2) to account for multiple comparisons (2 strata).

### Imputation analysis for ABO

We inferred the genotypes of the untyped SNPs in the *ABO* gene to estimate the *ABO* blood types. The typed SNPs in the *ABO* gene were pre-phased, and imputations were then performed using reference haplotypes from 286 East Asian subjects (CHB, 97; CHS, 100; and JPT, 89) from the 1000 Genomes Project March 2012 release (phase 1, version 3, http://brouser.1000genomes.org/). The ABO blood type of each individual was estimated using two major tagging SNPs, rs8176746(796C>A) and rs72238104(261delG), for the B- and O-type blood alleles, respectively.^16^ The imputed genotype at rs72238104 was used to determine the ABO blood type.

### Association analysis of imputed genotypes in FUT1 and FUT2 for ALP

We inferred the genotypes of the untyped, local SNPs around and within the *FUT1* and *FUT2* genes to assess other candidate SNPs in this region. The association of the imputed genotypes with the serum ALP level was analyzed using the same method described for the GWAS. The statistical level was set to 5×10^−8^.

### Software

The population structure was evaluated by principal component analysis using the software package EIGENSTRAT 3.0 (Harvard Medical School, Boston, MA, USA),^17^ and the GWAS was performed with PLINK 1.07 (http://pngu.mgh.harvard.edu/~purcell/plink/). A quantile–quantile plot (Q-Q plot) was generated using R 2.15.2 (http://cran.r-project.org/index.html). The Manhattan plot of −log_10_ *P* was depicted, and linkage disequilibrium (LD) analysis was performed using Haploview (http://www.broadinstitute.org/scientific-community/science/programs/medical-and-population-genetics/haploview/haploview). The regional plot was generated with ggplot2 (http://ggplot2.org/) or LocusZoom.^18^ The pre-phasing, imputation and association analyses of the imputed genotypes were performed with MaCH,^19^ Minimac^20^ and mach2qtl (http://www.sph.umich.edu/csg/abecasis/MACH/download/), respectively.

## Results

### SNPs and subjects for GWAS

A quality control assessment resulted in the exclusion of genotypes for 1,089,470 SNPs from the GWAS. The detailed SNP numbers were as follows: 65,681 non-autosomal SNPs, 17,919 SNPs with call rates <99%, 1,004,619 SNPs with MAFs <0.01 and 1,251 SNPs with Hardy–Weinberg equilibrium values of <5×10^−8^ (Fisher’s exact test). A total of 1,290,385 autosomal SNPs were subjected to GWAS. The log-transformed serum ALP values outside of the range of the mean±4 s.d. were excluded from the GWAS. The total number of serum ALP data points was 2,984.

### Genome-wide association analysis for ALP

**Figure 1:**
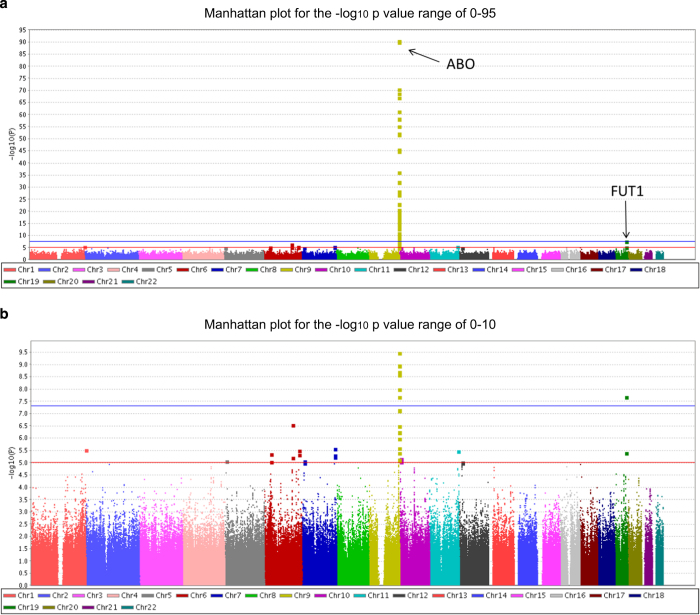
Manhattan plot of the genome-wide association analysis of alkaline phosphatase. The horizontal axis represents the chromosomal positions and the vertical axis depicts the −log_10_ *P* values from a test of association by linear regression analysis. The blue horizontal line represents a genome-wide significance *P* value of 5×10^−8^ and the red horizontal line corresponds to a *P* value of 1×10^−5^.

A GWAS of serum ALP levels was performed by linear regression analysis with the following five covariates: age, sex, BMI and the top two eigenvectors in principal component analysis. SNPs with a *P* value <1×10^−50^ on chromosome 9 and those with a *P* value <5×10^−8^ (the genome-wide significance level) on chromosomes other than chromosome 9 are shown in Table 1. All significant SNPs are shown in Supplementary Table 2. The Manhattan plot is shown in Figure 1. Q–Q plots of the GWAS indicated no evidence of genomic inflation (*λ*=1.007), as shown in Supplementary Figure 1. The SNP with the lowest *P* value was rs550057 in the *ABO* gene on 9q34.2 (*P*=2.15×10^−91^), and some other significant SNPs were located in the region around *ABO*. In a previous Japanese study, the primary SNP associated with serum ALP levels was rs657152 in *ABO*,^10^ and this finding was replicated in the present study (*P*=8.19×10^−68^). rs2071699 was also detected with an effect size of 1.0% and is located in the *FUT1* gene on chromosome 19q13.33. The statistical power for rs2071699 was calculated to be 50% from an effect size of 1.0% and a significance level of 5×10^−8^. No other significant SNPs were detected in this analysis. Regional association plots and LD blocks at the regions around and within the *ABO* and *FUT1* loci are presented in Supplementary Figures 2 and 3, respectively. SNPs in the *ABO* gene were separated into three LD blocks, and those with the three lowest *P* values (<1×10^−75^) were located in the most upstream LD block of the *ABO* gene. SNPs in the *FUT1* gene were located in one LD block of ~6 kb in size.

### Multiple SNP association analysis

To assess the mode of inheritance for the most significant SNPs in *ABO* and *FUT1* in the GWAS, linear regression analysis was performed with stepwise model selection using Akaike’s information criterion. Two significant SNPs of rs550057 in *ABO* and rs2071699 in *FUT1* were incorporated as genetic markers into a linear regression model with the covariates of age, sex and BMI. The mode of inheritance was determined to be additive for rs2071699 because the deviation of dominance was excluded, as shown in Supplementary Table 3. Furthermore, the coefficients for both additive and dominance for rs550057 had almost the same values, suggesting T allele as the dominant mode for rs550057.

### Gene–gene interaction analysis

We evaluated the effects of the interaction between rs2071699 in *FUT1* and rs550057 in *ABO* on serum ALP levels. As shown in Table 2, this interaction significantly affected the serum ALP levels. rs550057 in the *ABO* gene and rs2071699 in the *FUT1* gene accounted for 12.4 and 0.9% of the total variance in the serum ALP levels, respectively. The interaction between rs2071699 and rs550057 accounted for 0.3% of the total variance.

**Figure 2:**
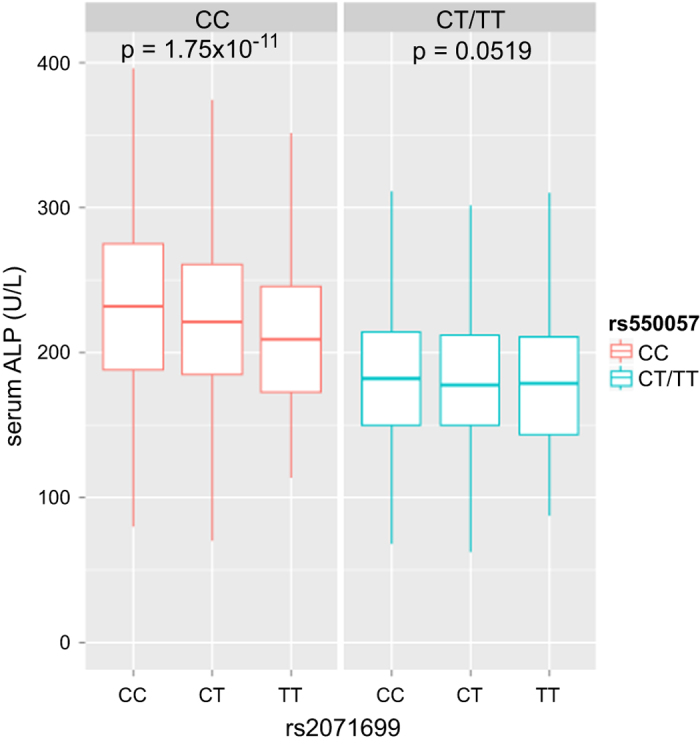
Box plots of serum alkaline phosphatase levels in groups categorized according to genotypes of rs550057 in *ABO* and rs2071699 in *FUT1.* The bottom, middle and top of each box represent the 25th, 50th and 75th percentiles for alkaline phosphatase levels, respectively. The lower and upper ends of each vertical bar represent the lower and upper adjacent values, respectively. The *P* value is shown for the rs2071699 genotypes in each stratum of the rs550057 genotype using a linear regression model for log_10_ alkaline phosphatase. The statistical level was set to 0.025 (0.05/2), accounting for the multiple comparisons.

In Figure 2, the serum ALP levels are shown for each group and are categorized according to the genotypes of both rs550057 and rs2071699. For the CC genotype carriers of rs550057 in *ABO*, the ALP levels decreased with increasing numbers of T alleles in rs2071699 of *FUT1*. However, the effects of the rs2071699 genotypes on ALP were negligible in carriers of the T allele of rs550057.

### Imputation analysis for ABO

The genotypes of the untyped SNPs in the *ABO* gene were inferred to estimate the ABO blood genotypes. The genotype at rs72238104 in the *ABO* gene was inferred in 2,977 out of 2,983 subjects. A comparison of the number of A-type blood alleles with rs550057 genotypes for each individual revealed that the number of these alleles coincided with the number of T alleles at rs550057 in more than 98% of the subjects (Table 3).

### Association analysis of imputed genotypes in FUT1 and FUT2 for ALP

**Figure 3:**
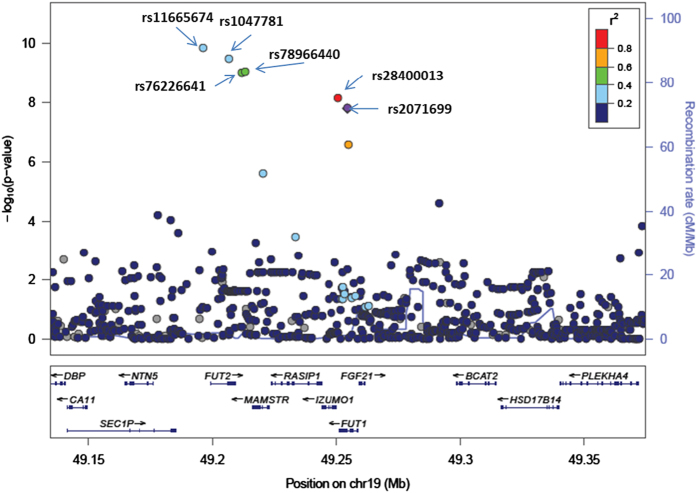
Regional association plot for imputed single-nucleotide polymorphism–alkaline phosphatase associations in the regions around and within the *FUT1* and *FUT2* loci. The *P* values of the imputed genotypes of the single-nucleotide polymorphisms in association analysis with serum alkaline phosphatase levels are plotted (as −log_10_ values) against their physical locations on chromosome 19. The estimated recombination rates in the Asian population of the 1000 Genomes Project (March 2012 release) show the local linkage disequilibrium structure. Each significant single-nucleotide polymorphism (*P* value<5×10^−8^) is indicated by an arrow. Inset: colors of other single-nucleotide polymorphisms indicate linkage disequilibrium with rs2071699 according to a scale from *r*^2^=0 to 1 based on pairwise *r*^2^ values from 1000 Genomes Project.

The association of serum ALP levels was analyzed using imputed genotypes around and within the *FUT1* and *FUT2* genes. The regional plot and the significant SNPs are shown in Figure 3 and Supplementary Table 4, respectively. Six SNPs around and within the *FUT1* and *FUT2* loci reached the established genome-wide significance level. Of these six SNPs, rs2071699 in the *FUT1* gene and rs1047781 in the *FUT2* gene were identified in the coding regions. rs1047781 is well known for its association with intestinal ALP levels in the serum of the Japanese population.^21^

## Discussion

The present study showed a strong association of SNPs in the *ABO* gene with serum ALP levels, and that the primary SNP was rs550057 in the *ABO* gene. The genotype at rs550057 was considered to be a determinant of the number of A-type blood alleles. Specifically, the number of T alleles at rs550057 was correlated with the number of A-type blood alleles in almost all individuals. The serum ALP levels were lower in the subjects with a T allele at rs550057 than in those without this allele, which is consistent with reports of lower-serum ALP levels in individuals with the A and AB ABO serotypes compared with the B and O ABO serotypes.

In the present GWAS, we detected a new marker in the *FUT1* gene that is associated with serum ALP levels in the healthy Japanese population. *FUT1* encodes α(1,2)-fucosyltransferase, which transfers a terminal fucose residue in an α(1,2)-linkage onto an existing galactose precursor molecule to form the H antigen, which is preferentially expressed in erythroid tissues and vascular endothelial cells.^22,23^ H antigen further undergoes specific sugar residue modification by glycosyltransferases that are encoded by *ABO*, resulting in the production of A or B antigens depending on the A- and B-type blood alleles located at the *ABO* locus. In contrast, the O-type blood allele does not produce an active enzyme.^24^ Thus, *FUT1* is closely linked to *ABO* in the production of ABH antigen on red blood cells. rs2071699, which was identified in this study, is a non-synonymous polymorphism in exon 3 of *FUT1* (35C>T, Ala12Val). The 35C>T SNP slightly reduces the activity of human α(1,2)-fucosyltransferase, as demonstrated by *in vitro* experiments using a mutant enzyme.^25^ This SNP may reduce the amount of ABH antigen on red blood cells, resulting in its reduced binding capacity for intestinal ALP in the serum. Thus, rs2071699 may affect serum ALP levels.

*FUT1* and *FUT2* are located within a 70-kb region on chromosome 19q. The *FUT2* gene is a determinant of secretor phenotypes^8^ that are associated with intestinal ALP levels in the serum.^4^ In the Japanese population, the secretor phenotype is determined primarily by rs1047781 in the *FUT2* gene, which is a missense mutation of 418A>T (Ile140Phe).^21^ This SNP almost completely reduces the activity of human α(1,2)-fucosyltransferase, as demonstrated by *in vitro* experiments using transfected cells.^20^ The negligible enzyme activity completely decreases the production of ABH antigen in gastric, intestinal, and salivary gland epithelial cells, as observed in individuals with the non-secretor phenotype. Although its mechanism is unknown, intestinal ALP levels in serum are detected in secretors, but not in non-secretors.

rs1047781 in the *FUT2* gene was not included in the DNA chip used in the present study, and no SNP in the *FUT2* gene was associated with serum ALP levels. However, rs1047781 was detected at the genome-wide significance level by association analysis using imputed genotypes. We also evaluated an interaction using imputed genotypes of rs1047781 with rs550057 in *ABO*, suggesting that the interaction with rs550057 in the *ABO* gene is significant and contributes to the interindividual variance of serum ALP levels, as well as rs2071699 in the *FUT1* gene (Supplementary Table 5). Furthermore, the LD between rs2071699 and rs1047781 was estimated to be moderate (*D*’=0.79, *r* ^2^=0.59) in Japanese samples obtained from the database of the 1000 Genomes Project. Thus, there is a high probability that rs2071699 is a marker of rs1047781.

The MAF of rs2071699 was 0.382 in the healthy Japanese population, which is comparable to that of JPT (0.360) in the HapMap database (http://hapmap.ncbi.nlm.nih.gov/index.html.en). Using the HapMap database, the MAFs of rs2071699 were 0.360, 0.244 and 0.022 in the Japanese, Chinese and European populations, respectively, indicating differences among populations. In Chinese and European studies, the *FUT1* locus has not been reported to be associated with serum ALP levels, which may be explained by the lower MAFs compared with the Japanese population.

The effects of the rs2071699 genotypes in *FUT1* on serum ALP levels were negligible in the carriers of the T allele (CT/TT genotype) of rs550057 in *ABO*. However, serum ALP levels decreased with increasing numbers of T alleles of rs2071699 in *FUT1* in non-carriers of the T allele (CC genotype) of rs550057 in *ABO*. This result suggests that the *FUT1* and/or *FUT2* genes interact with the *ABO* gene to affect serum ALP levels. Comparing the genotype groups, the serum ALP level was 30% higher in the group with the highest ALP level compared with that with the lowest level (CC at rs2071699 and CC at rs550057 versus TT at rs2071699 and CT or TT at rs550057), as shown in Figure 2. These results suggest the importance of considering these genotypes in establishing the normal range of ALP in the clinical setting.

In summary, we detected SNP markers of rs550057 in the *ABO* gene and rs2071699 in the *FUT1* gene in association with serum ALP levels by GWAS in a healthy Japanese population. Further association analysis using imputed genotypes detected rs1047781 in the *FUT2* gene well known in the association with serum ALP levels. Investigation for the interaction between *ABO* and *FUT1*/*FUT2* suggested that there is evidence of interaction between *ABO* and *FUT1*/*FUT2* on serum ALP levels, regardless of the possibility that rs2071699 in *FUT1* is a proxy of rs1047781 in *FUT2*.

## Figures and Tables

**Table 1:** SNPs associated with serum ALP levels

*SNP ID*	*Chromosome*	*Position*^a^	*Gene*	*Location*	*Minor/major allele*	*Minor allele frequency*	P *value*^b^
rs550057	9	136146597	ABO	intron	T/C	0.274	2.15×10^−91^
rs532436	9	136149830	ABO	intron	A/G	0.275	6.43×10^−91^
rs507666	9	136149399	ABO	intron	A/G	0.275	6.43×10^−91^
rs579459	9	136154168	ABO|SURF6	intergenic	C/T	0.275	7.12×10^−91^
rs7849280	9	136126636	LCN1L1|ABO	intergenic	G/A	0.251	1.93×10^−71^
rs7025839	9	136124190	LCN1L1|ABO	intergenic	A/G	0.248	1.23×10^−69^
rs657152	9	136139265	ABO	intron	T/G	0.431	8.19×10^−68^
rs687289	9	136137106	ABO	intron	T/C	0.445	3.62×10^−62^
rs186775362	9	136149096	ABO	intron	C/A	0.442	2.95×10^−59^
rs529565	9	136149500	ABO	intron	C/T	0.442	3.97×10^−59^
rs9411475	9	136127268	LCN1L1|ABO	intergenic	C/T	0.298	5.65×10^−56^
rs9919007	9	136119527	LCN1L1|ABO	intergenic	T/C	0.399	4.89×10^−53^
rs9411468	9	136119888	LCN1L1|ABO	intergenic	A/G	0.398	1.41×10^−52^
rs2071699	19	49254504	FUT1	coding	T/C	0.382	1.99×10^−08^

Abbreviations: ALP, alkaline phosphatase; BMI, body mass index; PCA, priciple component analysis; SNP, single-nucleotide polymorphism.

SNPs with a *P* value <1×10^−50^ on chromosome 9 and those with a *P* value <the genome-wide significance level of 5×10^−8^ on chromosomes other than chromosome 9 are shown.

a Genome position is based on NCBI build 37.1.

b The *P* values in the GWAS stage are based on linear regression analysis of the log-transformed ALP values with adjustments for age, sex, BMI and the top two eigenvectors in PCA analysis, assuming an additive model by PLINK.

**Table 2:** Gene–gene interaction analysis of serum ALP levels followed by analysis of variance

	*Estimate*	*s.e.*	t *value*	P *value (>|t|)*
*(A) Linear multiple regression*				
(Intercept)	5.314	0.0375	141.52	<2.00×10^−16^
Age	0.002	0.0004	3.82	1.35×10^−04^
Sex	−0.179	0.0099	−18.18	<2.00×10^−16^
BMI	0.007	0.0015	4.58	4.83×10^−06^
rs550057	−0.241	0.0138	−17.54	<2.00×10^−16^
rs2071699	−0.063	0.0094	−6.72	2.10×10^11^
rs550057:rs2071699	0.045	0.0133	3.37	7.64×10^−04^

	*Degree of freedom*	*Sum of squares*	*Mean squares*	F *value*	P *value (>F)*	*Variance explained*
*(B) Analysis of variance*						
Age	1	3.41	3.41	53.6	3.14×10^−13^	0.013
Sex	1	28.41	28.41	446.4	<2.20×10^−16^	0.110
BMI	1	1.32	1.32	20.7	5.49×10^−06^	0.005
rs550057	1	31.80	31.80	499.6	<2.20×10^−16^	0.124
rs2071699	1	2.39	2.39	37.5	1.02×10^−09^	0.009
rs550057:rs2071699	1	0.72	0.72	11.4	7.64×10^−04^	0.003
Residuals	2,976	189.41	0.06	—	—	0.736

Abbreviations: ALP, alkaline phosphatase; BMI, body mass index.

(A) Regression analysis of log-transformed serum ALP levels with the covariates age, sex, BMI, rs550057, rs2071699 and the interaction term between rs550057 and rs2071699 (as shown as rs550057:rs2071699). The mode of inheritance was assumed as T-allele dominant for rs550057 and as an additive for rs2071699.

(B) The explained variance was calculated as the proportion of the variance of the log-transformed serum ALP levels divided by the variable.

**Table 3:** The relationship between rs550057 genotype and number of A-type blood alleles estimated from imputation

*Number of A-type blood alleles*	*ABO genotype*^a^	n	*rs550057*		
			*T/T*	*C/T*	*C/C*
2	AA	243	232	11	0
			(100%)	(0.9%)	(0%)
1	AO	880	0	1152	29
	AB	301	(0%)	(98.9%)	(1.8%)
0	OO	934	0	2	1553
	BO	549	(0%)	(0.2%)	(98.2%)
	BB	72			
Total			233	1,164	1,580

Each figure in parentheses indicates the percentage of subjects in each category with the A-type blood allele relative to the number of subjects with each genotype at rs550057.

a The blood type was estimated from the genotypes at two tag-SNPs, rs8176746 and rs72238104, which are responsible for the B- and O-type blood alleles, respectively. The ABO blood genotype was categorized by the number of A-type blood alleles.

## Notes

The authors are employees of Otsuka Pharmaceuticals.
