# Supplementary material for: Interaction of genetic markers associated with serum alkaline phosphatase levels in the Japanese population
Source: Hum Genome Var. 2015 Jul 2;2:15019–. doi: 10.1038/hgv.2015.19 (PMC4785570; doi:10.1038/hgv.2015.19)
Supplement: Supplementary Table 1 [file hgv201519-s1.doc]

## Supplemental Table 1 - Characteristics of subjects for GWAS

| Characteristic | Male (n=1590) | Female (n=1394) |
| --- | --- | --- |
| Age (years) | 36.0±11.3 | 34.2±10.2 |
| Height (cm) | 171.1±5.9 | 158.3±5.3 |
| Body weight (kg) | 67.8±10.6 | 52.3±7.7 |
| Body mass index (kg/m^2^) | 23.1±3.2 | 20.9±2.9 |
|  |  |  |
| ALP (U/L) | 227±59 | 188±59 |

Each figure represents the mean ± SD.
