# Supplementary material for: Interaction of genetic markers associated with serum alkaline phosphatase levels in the Japanese population
Source: Hum Genome Var. 2015 Jul 2;2:15019–. doi: 10.1038/hgv.2015.19 (PMC4785570; doi:10.1038/hgv.2015.19)
Supplement: Supplementary Table 2 [file hgv201519-s2.doc]

## Supplemental Table 2 - SNPs associated with serum ALP levels

| SNP ID | Chromosome | Position^a^ | Gene | Location | Minor/Major Allele | Minor Allele Frequency | BETA^b^ | SE | p value^c^ |
| --- | --- | --- | --- | --- | --- | --- | --- | --- | --- |
| rs550057 | 9 | 136146597 | ABO | intron | T/C | 0.274 | -0.155 | 0.00739 | 2.15×10^-91^ |
| rs532436 | 9 | 136149830 | ABO | intron | A/G | 0.275 | -0.155 | 0.00739 | 6.43×10^-91^ |
| rs507666 | 9 | 136149399 | ABO | intron | A/G | 0.275 | -0.155 | 0.00739 | 6.43×10^-91^ |
| rs579459 | 9 | 136154168 | ABO | SURF6 | intergenic | C/T | 0.275 | -0.155 | 0.00739 | 7.12×10^-91^ |
| rs7849280 | 9 | 136126636 | LCN1L1 | ABO | intergenic | G/A | 0.251 | -0.143 | 0.00781 | 1.93×10^-71^ |
| rs7025839 | 9 | 136124190 | LCN1L1 | ABO | intergenic | A/G | 0.248 | -0.142 | 0.00782 | 1.23×10^-69^ |
| rs657152 | 9 | 136139265 | ABO | intron | T/G | 0.431 | -0.120 | 0.00675 | 8.19×10^-68^ |
| rs687289 | 9 | 136137106 | ABO | intron | T/C | 0.445 | -0.115 | 0.00673 | 3.62×10^-62^ |
| rs186775362 | 9 | 136149096 | ABO | intron | C/A | 0.442 | -0.112 | 0.00673 | 2.95×10^-59^ |
| rs529565 | 9 | 136149500 | ABO | intron | C/T | 0.442 | -0.112 | 0.00673 | 3.97×10^-59^ |
| rs9411475 | 9 | 136127268 | LCN1L1 | ABO | intergenic | C/T | 0.298 | -0.120 | 0.00744 | 5.65×10^-56^ |
| rs9919007 | 9 | 136119527 | LCN1L1 | ABO | intergenic | T/C | 0.399 | -0.108 | 0.00690 | 4.89×10^-53^ |
| rs9411468 | 9 | 136119888 | LCN1L1 | ABO | intergenic | A/G | 0.398 | -0.108 | 0.00692 | 1.41×10^-52^ |
| rs9411367 | 9 | 136118513 | LCN1L1 | ABO | intergenic | T/C | 0.397 | -0.101 | 0.00696 | 2.11×10^-46^ |
| rs633862 | 9 | 136155444 | ABO | SURF6 | intergenic | G/A | 0.476 | 0.099 | 0.00683 | 1.09×10^-45^ |
| rs7470777 | 9 | 136115176 | LCN1L1 | ABO | intergenic | G/A | 0.322 | -0.096 | 0.00744 | 4.30×10^37^ |
| rs4962111 | 9 | 136112537 | LCN1L1 | ABO | intergenic | C/T | 0.365 | -0.087 | 0.00720 | 3.06×10^-33^ |
| rs4357365 | 9 | 136113834 | LCN1L1 | ABO | intergenic | C/G | 0.373 | -0.087 | 0.00717 | 5.76×10^-33^ |
| rs630014 | 9 | 136149722 | ABO | intron | T/C | 0.372 | 0.082 | 0.00726 | 4.29×10^-29^ |
| rs4962113 | 9 | 136127641 | LCN1L1 | ABO | intergenic | C/T | 0.377 | 0.081 | 0.00715 | 4.90×10^29^ |
| rs9411463 | 9 | 136105515 | LCN1L1 | ABO | intergenic | T/C | 0.302 | -0.084 | 0.00764 | 1.02×10^-27^ |
| rs4962043 | 9 | 136177993 | ABO | SURF6 | intergenic | A/G | 0.396 | 0.073 | 0.00717 | 5.73×10^-24^ |
| rs12378537 | 9 | 136065526 | GBGT1 | OBP2B | intergenic | T/C | 0.270 | -0.081 | 0.00798 | 9.62×10^-24^ |
| rs12554580 | 9 | 136128603 | LCN1L1 | ABO | intergenic | C/T | 0.429 | 0.068 | 0.00711 | 1.49×10^-21^ |
| rs672316 | 9 | 136138125 | ABO | intron | C/A | 0.272 | 0.076 | 0.00791 | 1.87×10^-21^ |
| rs641959 | 9 | 136133699 | ABO | intron | G/T | 0.272 | 0.075 | 0.00789 | 2.43×10^-21^ |
| rs514708 | 9 | 136133743 | ABO | intron | T/C | 0.271 | 0.075 | 0.00790 | 3.32×10^-21^ |
| rs8176682 | 9 | 136139297 | ABO | intron | A/G | 0.297 | 0.072 | 0.00757 | 5.11×10^-21^ |
| rs10901246 | 9 | 136080485 | GBGT1 | OBP2B | intergenic | G/T | 0.407 | -0.067 | 0.00712 | 5.57×10^-21^ |
| rs8176681 | 9 | 136139754 | ABO | intron | G/A | 0.297 | 0.071 | 0.00757 | 7.35×10^-21^ |
| rs10901245 | 9 | 136079654 | GBGT1 | OBP2B | intergenic | T/C | 0.408 | -0.067 | 0.00712 | 8.46×10^-21^ |
| rs10901244 | 9 | 136079463 | GBGT1 | OBP2B | intergenic | C/T | 0.408 | -0.067 | 0.00712 | 8.46×10^-21^ |
| rs4962103 | 9 | 136077516 | GBGT1 | OBP2B | intergenic | G/A | 0.408 | -0.067 | 0.00712 | 8.51×10^-21^ |
| rs35919591 | 9 | 136078652 | GBGT1 | OBP2B | intergenic | A/G | 0.408 | -0.067 | 0.00712 | 8.69×10^-21^ |
| rs512770 | 9 | 136133506 | ABO | coding | A/G | 0.258 | 0.074 | 0.00805 | 3.86×10^-20^ |
| rs549443 | 9 | 136135237 | ABO | coding | A/G | 0.260 | 0.074 | 0.00800 | 4.60×10^20^ |
| rs549446 | 9 | 136135238 | ABO | coding | T/C | 0.260 | 0.074 | 0.00799 | 5.82×10^-20^ |
| rs574347 | 9 | 136135659 | ABO | intron | C/T | 0.260 | 0.074 | 0.00800 | 6.18×10^-20^ |
| rs688976 | 9 | 136136770 | ABO | coding | T/G | 0.260 | 0.073 | 0.00799 | 7.21×10^-20^ |
| rs8176732 | 9 | 136132303 | ABO | intron | C/T | 0.260 | 0.073 | 0.00801 | 8.52×10^-20^ |
| rs62574565 | 9 | 136128259 | LCN1L1 | ABO | intergenic | A/G | 0.269 | 0.073 | 0.00798 | 1.79×10^-19^ |
| rs8176740 | 9 | 136131472 | ABO | coding | A/T | 0.263 | 0.073 | 0.00806 | 2.15×10^-19^ |
| rs474279 | 9 | 136139617 | ABO | intron | T/C | 0.255 | 0.072 | 0.00806 | 5.03×10^-19^ |
| rs886082 | 9 | 136101999 | LCN1L1 | intron | T/G | 0.456 | -0.063 | 0.00708 | 1.13×10^-18^ |
| rs568203 | 9 | 136151445 | ABO | SURF6 | intergenic | A/C | 0.264 | 0.070 | 0.00792 | 1.49×10^-18^ |
| rs11244041 | 9 | 136109279 | LCN1L1 | ABO | intergenic | G/A | 0.460 | -0.062 | 0.00705 | 2.03×10^-18^ |
| rs552148 | 9 | 136153481 | ABO | SURF6 | intergenic | T/C | 0.265 | 0.070 | 0.00793 | 2.42×10^-18^ |
| rs7857390 | 9 | 136128546 | LCN1L1 | ABO | intergenic | A/G | 0.286 | 0.067 | 0.00763 | 2.54×10^-18^ |
| rs4962127 | 9 | 136178277 | ABO | SURF6 | intergenic | A/G | 0.266 | 0.069 | 0.00790 | 3.04×10^-18^ |
| rs624960 | 9 | 136175051 | ABO | SURF6 | intergenic | T/C | 0.267 | 0.069 | 0.00789 | 3.73×10^-18^ |
| rs1752337 | 9 | 136160228 | ABO | SURF6 | intergenic | C/A | 0.266 | 0.068 | 0.00789 | 1.73×10^-17^ |
| rs9411394 | 9 | 136182391 | ABO | SURF6 | intergenic | T/G | 0.270 | 0.064 | 0.00788 | 4.66×10^-16^ |
| rs7864821 | 9 | 136086916 | OBP2B | LCN1L1 | intergenic | C/T | 0.355 | -0.059 | 0.00743 | 4.81×10^-15^ |
| rs3761821 | 9 | 136085783 | OBP2B | LCN1L1 | intergenic | T/C | 0.353 | -0.059 | 0.00745 | 4.81×10^-15^ |
| rs11244034 | 9 | 136080272 | GBGT1 | OBP2B | intergenic | T/C | 0.359 | -0.058 | 0.00738 | 5.10×10^15^ |
| rs4507838 | 9 | 136102199 | LCN1L1 | UTR | A/G | 0.390 | -0.057 | 0.00727 | 8.76×10^-15^ |
| rs4962125 | 9 | 136176977 | ABO | SURF6 | intergenic | C/T | 0.471 | 0.055 | 0.00709 | 1.39×10^-14^ |
| rs3124752 | 9 | 136264493 | C9orf96 | intron | G/A | 0.311 | -0.058 | 0.00752 | 1.68×10^-14^ |
| rs9411381 | 9 | 136156067 | ABO | SURF6 | intergenic | C/T | 0.200 | 0.068 | 0.00890 | 2.23×10^-14^ |
| rs3124782 | 9 | 136270996 | C9orf96 / REXO4 | COMPLEX | G/A | 0.327 | -0.055 | 0.00737 | 9.80×10^14^ |
| rs8176635 | 9 | 136152009 | ABO | SURF6 | intergenic | T/C | 0.353 | 0.056 | 0.00751 | 1.12×10^-13^ |
| rs12335 | 9 | 136197834 | SURF6 | UTR | G/A | 0.336 | -0.051 | 0.00734 | 7.11×10^-12^ |
| rs886090 | 9 | 136199503 | SURF6 | coding | A/G | 0.320 | -0.051 | 0.00749 | 7.47×10^-12^ |
| rs3124781 | 9 | 136275313 | REXO4 | intron | A/G | 0.300 | -0.050 | 0.00758 | 4.89×10^-11^ |
| rs3124779 | 9 | 136281966 | REXO4 | intron | T/C | 0.301 | -0.050 | 0.00758 | 7.33×10^-11^ |
| rs8176694 | 9 | 136137646 | ABO | intron | G/A | 0.116 | 0.070 | 0.01104 | 3.22×10^-10^ |
| rs1059773 | 9 | 136228865 | SURF4 | UTR | T/G | 0.307 | -0.046 | 0.00758 | 1.05×10^-9^ |
| rs12763 | 9 | 136227260 | SURF2 | coding | A/G | 0.307 | -0.046 | 0.00758 | 1.05×10^-9^ |
| rs553877 | 9 | 136214955 | MED22 | UTR | A/G | 0.306 | -0.046 | 0.00758 | 1.10×10^9^ |
| rs4962046 | 9 | 136237783 | SURF4 | intron | A/C | 0.214 | 0.052 | 0.00860 | 1.87×10^-9^ |
| rs886089 | 9 | 136199466 | SURF6 | coding | A/G | 0.309 | -0.045 | 0.00756 | 2.09×10^-9^ |
| rs17150482 | 9 | 136194595 | ABO | SURF6 | intergenic | C/T | 0.309 | -0.045 | 0.00755 | 2.28×10^-9^ |
| rs10751505 | 9 | 136195374 | ABO | SURF6 | intergenic | G/A | 0.309 | -0.045 | 0.00756 | 2.53×10^-9^ |
| rs2039184 | 9 | 136047393 | GBGT1 | OBP2B | intergenic | C/T | 0.275 | -0.046 | 0.00801 | 9.61×10^-9^ |
| rs11244030 | 9 | 136075549 | GBGT1 | OBP2B | intergenic | T/C | 0.428 | 0.040 | 0.00714 | 1.99×10^-8^ |
| rs2071699 | 19 | 49254504 | FUT1 | coding | T/C | 0.382 | -0.041 | 0.00723 | 1.99×10^-8^ |

^a^The genome position is based on NCBI build 37.1.

^b^The regression coefficient in the GWAS stage is based on linear regression analysis of the log-transformed ALP values with adjustments for age, sex, BMI, and the top two eigenvectors in PCA analysis, assuming an additive model by PLINK.

^c^The p values in the GWAS stage.
