# Supplementary material for: Interaction of genetic markers associated with serum alkaline phosphatase levels in the Japanese population
Source: Hum Genome Var. 2015 Jul 2;2:15019–. doi: 10.1038/hgv.2015.19 (PMC4785570; doi:10.1038/hgv.2015.19)
Supplement: Supplementary Table 3 [file hgv201519-s3.doc]

**Supplemental Table 3** - Linear multiple regression analysis of serum ALP levels followed by analysis of variance

| (A) Linear multiple regression | |  |  |  |  |  |
| --- | --- | --- | --- | --- | --- | --- |
|  | Estimate | SE | t value | p value(>|t|) |  |  |
| (Intercept) | 5.117 | 0.0341 | 149.94 | <2.00×10^-16^ |  |  |
| AGE | 0.002 | 0.0004 | 3.63 | 2.85×10^-04^ |  |  |
| SEX | 0.179 | 0.0099 | 18.16 | <2.00×10^-16^ |  |  |
| BMI | 0.007 | 0.0015 | 4.68 | 2.99×10^-06^ |  |  |
| rs550057_additive | -0.118 | 0.0089 | -13.36 | <2.00×10^-16^ |  |  |
| rs550057_dom | -0.083 | 0.0115 | -7.18 | 8.79×10^-13^ |  |  |
| rs2071699_additive | -0.041 | 0.0067 | -6.08 | 1.40×10^-09^ |  |  |
|  |  |  |  |  |  |  |
|  |  |  |  |  |  |  |
| (B) Analysis of variance |  |  |  |  |  |  |
|  | Degree of freedom | Sum of squares | Mean squares | F value | p value(>F) | Variance explained |
| AGE | 1 | 3.41 | 3.41 | 53.5 | 3.36×10^-13^ | 0.013 |
| SEX | 1 | 28.41 | 28.41 | 445.3 | <2.20×10^-16^ | 0.110 |
| BMI | 1 | 1.32 | 1.32 | 20.7 | 5.64×10^-06^ | 0.005 |
| rs550057_additive | 1 | 28.89 | 28.89 | 452.8 | <2.20×10^-16^ | 0.112 |
| rs550057_dom | 1 | 3.19 | 3.18 | 49.9 | 1.99×10^-12^ | 0.012 |
| rs2071699_additive | 1 | 2.36 | 2.35 | 36.9 | 1.40×10^-09^ | 0.009 |
| Residuals | 2976 | 189.89 | 0.06 |  |  | 0.738 |

(A) Regression analysis with stepwise model selection was performed and the selected model is shown. For each SNP, additive effect is shown as the dbSNP ID, and dominance deviation is shown as dbSNP ID_dom.

(B) The explained variance was calculated as the proportion of the variance of the log-transformed serum ALP levels divided by the variable.
