# Supplementary material for: Interaction of genetic markers associated with serum alkaline phosphatase levels in the Japanese population
Source: Hum Genome Var. 2015 Jul 2;2:15019–. doi: 10.1038/hgv.2015.19 (PMC4785570; doi:10.1038/hgv.2015.19)
Supplement: Supplementary Table 4 [file hgv201519-s4.doc]

## Supplemental Table 4 - SNPs associated with serum ALP levels in association analysis of imputed genotypes around and within *FUT1* and *FUT2* loci

| SNP ID | Chromosome | Position^a^ | Gene | Location | Minor/Major Allele | Minor Allele Frequency | BETA^b^ | SE | p value |
| --- | --- | --- | --- | --- | --- | --- | --- | --- | --- |
| rs11665674 | 19 | 49196275 | SEC1P | FUT2 | intergenic | G/A | 0.4685 | -0.059 | 0.009 | 1.38×10^-10^ |
| rs1047781 | 19 | 49206631 | FUT2 | coding | T/A | 0.4237 | -0.057 | 0.009 | 3.26×10^-10^ |
| rs78966440 | 19 | 49213154 | FUT2 | MAMSTR | intergenic | C/T | 0.4578 | -0.054 | 0.009 | 8.69×10^-10^ |
| rs76226641 | 19 | 49211832 | FUT2 | MAMSTR | intergenic | C/G | 0.4587 | -0.054 | 0.009 | 9.38×10^-10^ |
| rs28400013 | 19 | 49250672 | IZMO1 | UTR | T/C | 0.3957 | -0.045 | 0.008 | 7.30×10^-9^ |
| rs2071699 | 19 | 49254504 | FUT1 | coding | A/G | 0.3825 | -0.041 | 0.007 | 1.59×10^-8^ |

^a^The genome position is based on NCBI build 37.1.

^b^The regression coefficient is based on linear regression analysis of the log-transformed ALP values with adjustments for age, sex, BMI, and the top two eigenvectors in PCA analysis, assuming an additive model by mach2qtl.
