# Supplementary material for: Interaction of genetic markers associated with serum alkaline phosphatase levels in the Japanese population
Source: Hum Genome Var. 2015 Jul 2;2:15019–. doi: 10.1038/hgv.2015.19 (PMC4785570; doi:10.1038/hgv.2015.19)
Supplement: Supplementary Table 5 [file hgv201519-s5.doc]

**Supplemental Table 5 - Gene-gene interaction analysis of serum ALP levels followed by analysis of variance using imputed genotypes of rs1047781 in *FUT2***

| (A) Linear multiple regression | |  |  |  |  |  |
| --- | --- | --- | --- | --- | --- | --- |
|  | Estimate | SE | t value | p value(>|t|) |  |  |
| (Intercept) | 5.327 | 0.0376 | 141.75 | <2.00×10^-16^ |  |  |
| AGE | 0.002 | 0.0004 | 3.74 | 1.90×10^-04^ |  |  |
| SEX | -0.180 | 0.0098 | -18.27 | <2.00×10^-16^ |  |  |
| BMI | 0.007 | 0.0015 | 4.60 | 4.40×10^-06^ |  |  |
| rs550057 | -0.248 | 0.0144 | -17.18 | <2.00×10^-16^ |  |  |
| rs1047781 | -0.073 | 0.0096 | -7.61 | 3.57×10^-14^ |  |  |
| rs550057:rs1047781 | 0.050 | 0.0137 | 3.65 | 2.63×10^-04^ |  |  |
|  |  |  |  |  |  |  |
| (B) Analysis of variance |  |  |  |  |  |  |
|  | Degree of freedom | Sum of squares | Mean squares | F value | p value(>F) | Variance explained |
| AGE | 1 | 3.42 | 3.42 | 54.1 | 2.50×10^-13^ | 0.013 |
| SEX | 1 | 28.42 | 28.42 | 448.8 | <2.20×10^-16^ | 0.110 |
| BMI | 1 | 1.32 | 1.32 | 20.9 | 5.15×10^-06^ | 0.005 |
| rs550057 | 1 | 31.79 | 31.79 | 501.9 | <2.20×10^-16^ | 0.123 |
| rs1047781 | 1 | 3.16 | 3.16 | 50.0 | 1.95×10^-12^ | 0.012 |
| rs550057:rs1047781 | 1 | 0.85 | 0.85 | 13.4 | 2.63×10^-04^ | 0.003 |
| Residuals | 2977 | 188.52 | 0.06 |  |  | 0.732 |

(A) Regression analysis of log-transformed serum ALP levels with the covariates age, sex, BMI, rs550057, rs1047781, and the interaction term between rs550057 and rs1047781 (as shown as rs550057:rs1047781). The mode of inheritance was assumed as T allele dominant for rs550057 and as additive for rs1047781.

(B) The explained variance was calculated as the proportion of the variance of the log-transformed serum ALP levels divided by the variable.
