# Supplementary material for: Interaction of genetic markers associated with serum alkaline phosphatase levels in the Japanese population
Source: Hum Genome Var. 2015 Jul 2;2:15019–. doi: 10.1038/hgv.2015.19 (PMC4785570; doi:10.1038/hgv.2015.19)
Supplement: Supplementary Figure 1 [file hgv201519-s6.doc]

| 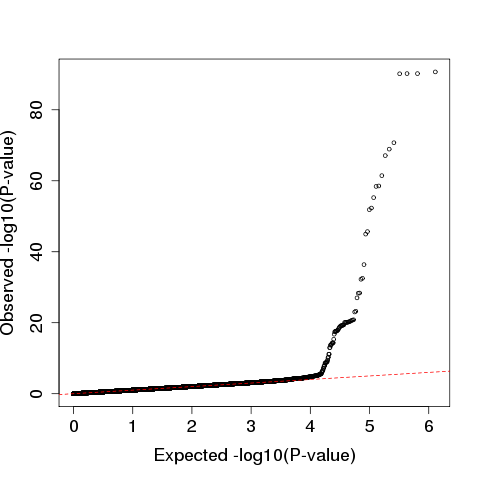 |
| --- |

## Supplemental Figure 1 - Q-Q plots of GWAS for ALP

The horizontal axis shows the expected -log_10_-transformed p values, and the vertical axis indicates the observed -log_10_-transformed p values. The genomic inflation factor, λ, is 1.007.
