# Supplementary material for: Interaction of genetic markers associated with serum alkaline phosphatase levels in the Japanese population
Source: Hum Genome Var. 2015 Jul 2;2:15019–. doi: 10.1038/hgv.2015.19 (PMC4785570; doi:10.1038/hgv.2015.19)
Supplement: Supplementary Figure 2 [file hgv201519-s7.doc]

| 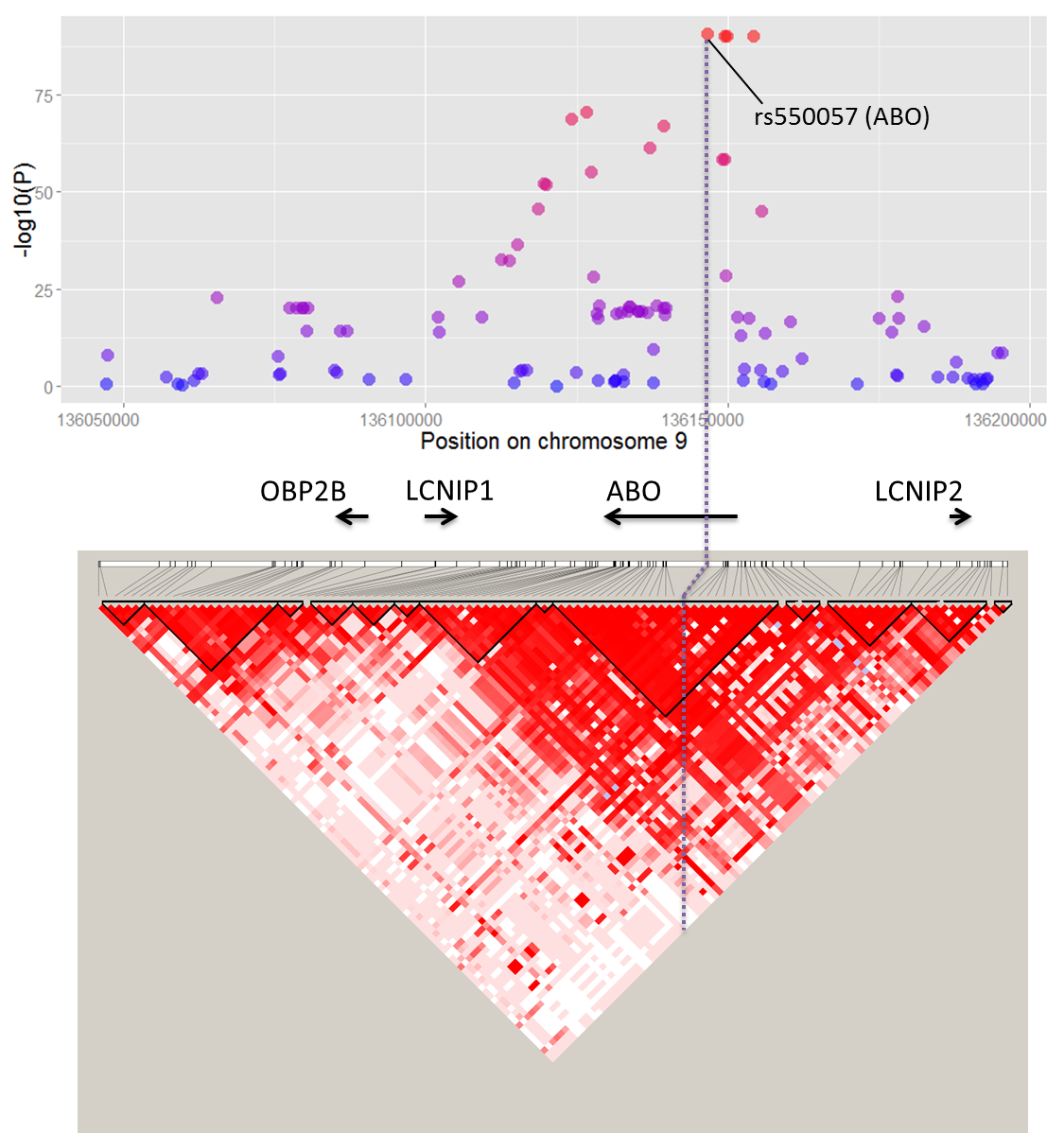 |
| --- |

## Supplemental Figure 2 - Regional association plot and LD block for SNP-ALP association at the region around and within the *ABO* locus

## (A) Regional association plot

## The horizontal axis shows the chromosomal positions in the NCBI build 37.1 genome sequences. Each dot represents a -log_10_ p value of an SNP genotyped using Illumina HumanOmni 2.5-8.

## (B) LD block

The LD structure of the region around *ABO* is shown. The LD heatmap indicates higher D' values, depicted as darker red colors.
