# Supplementary material for: Interaction of genetic markers associated with serum alkaline phosphatase levels in the Japanese population
Source: Hum Genome Var. 2015 Jul 2;2:15019–. doi: 10.1038/hgv.2015.19 (PMC4785570; doi:10.1038/hgv.2015.19)
Supplement: Supplementary Information [file hgv201519-s9.doc]

# Supplementary Information

This document is the brief instructions on how to use the supplemental information.

## Supplemental Table 1 - Characteristics of subjects for GWAS

Each figure represents the mean ± SD.

## Supplemental Table 2 - SNPs associated with serum ALP levels

^a^The genome position is based on NCBI build 37.1.

^b^The regression coefficient in the GWAS stage is based on linear regression analysis of the log-transformed ALP values with adjustments for age, sex, BMI, and the top two eigenvectors in PCA analysis, assuming an additive model by PLINK.

^c^The p values in the GWAS stage.

## Supplemental Table 3 - Linear multiple regression analysis of serum ALP levels followed by analysis of variance

(A) Regression analysis with stepwise model selection was performed and the selected model is shown. For each SNP, additive effect is shown as the dbSNP ID, and dominance deviation is shown as dbSNP ID_dom.

(B) The explained variance was calculated as the proportion of the variance of the log-transformed serum ALP levels divided by the variable.

## Supplemental Table 4 - SNPs associated with serum ALP levels in association analysis of imputed genotypes around and within *FUT1* and *FUT2* loci

^a^The genome position is based on NCBI build 37.1.

^b^The regression coefficient is based on linear regression analysis of the log-transformed ALP values with adjustments for age, sex, BMI, and the top two eigenvectors in PCA analysis, assuming an additive model by mach2qtl.

Supplemental Table 5 - Gene-gene interaction analysis of serum ALP levels followed by analysis of variance using imputed genotypes of rs1047781 in *FUT2*

(A) Regression analysis of log-transformed serum ALP levels with the covariates age, sex, BMI, rs550057, rs1047781, and the interaction term between rs550057 and rs1047781 (as shown as rs550057:rs1047781). The mode of inheritance was assumed as T allele dominant for rs550057 and as additive for rs1047781.

(B) The explained variance was calculated as the proportion of the variance of the log-transformed serum ALP levels divided by the variable.

## Supplemental Figure 1 - Q-Q plots of GWAS for ALP

The horizontal axis shows the expected -log_10_-transformed p values, and the vertical axis indicates the observed -log_10_-transformed p values. The genomic inflation factor, λ, is 1.007.

## Supplemental Figure 2 - Regional association plot and LD block for SNP-ALP association at the region around and within the *ABO* locus

## (A) Regional association plot

## The horizontal axis shows the chromosomal positions in the NCBI build 37.1 genome sequences. Each dot represents a -log_10_ p value of an SNP genotyped using Illumina HumanOmni 2.5-8.

## (B) LD block

The LD structure of the region around *ABO* is shown. The LD heatmap indicates higher D' values, depicted as darker red colors.

## Supplemental Figure 3 - Regional association plot and LD block for SNP-ALP association at the region around and within the *FUT1* locus

## (A) Regional association plot

## The horizontal axis shows the chromosomal positions in the NCBI build 37.1 genome sequences. Each dot represents a -log_10_ p value of an SNP genotyped by Illumina HumanOmni 2.5-8.

## (B) LD block

The LD structure of the region around *FUT1* is shown. The LD heatmap indicates higher D' values depicted as darker red colors.
